# The relationship in women between genetic liability and the risk of onset of alcohol use disorder while pregnant or rearing an infant, toddler, or preschool child

**DOI:** 10.1038/s41380-025-03410-5

**Published:** 2025-12-14

**Authors:** Kenneth S. Kendler, Dace Svikis, Linda Abrahamsson, Jan Sundquist, Kristina Sundquist

**Affiliations:** 1 Virginia Institute for Psychiatric and Behavioral Genetics, Virginia Commonwealth University, Richmond, VA USA; 2 Department of Psychiatry, Virginia Commonwealth University, Richmond, VA USA; 3 Department of Psychology, Virginia Commonwealth University, Richmond, VA USA; 4 Center for Primary Health Care Research, Lund University, Malmö, Sweden

**Keywords:** Genetics, Psychology

## Abstract

While pregnancy clearly reduces the risk for Alcohol Use Disorder (AUD) onset, we know less about the impact on AUD risk of having young children and how these effects vary across maternal age and level of maternal AUD genetic risk. Therefore, in 1.2 million parous Swedish women born 1960–1995, we examined those with a first registration for AUD between ages 15–40 while first pregnant, or while raising their first infant (aged 0–12 months), toddler (13–36 months) or preschooler (37–60 months). Genetic risk for AUD was assessed by their family genetic risk score. Pregnancy and having an infant consistently reduced AUD risk with the protective effect becoming stronger with increasing maternal genetic risk. Raising a toddler was modestly protective, but unrelated to genetic risk. Raising a preschooler, while unrelated to AUD risk in mothers with low genetic liability, in those at higher genetic liability increased AUD risk considerably. These effects varied substantially across maternal age. Being pregnant or having an infant were only marginally protective in teenage mothers. Compared to older mothers, younger mothers were considerably more sensitive to the predisposing effects on AUD risk of toddlers and preschoolers. The effects of pregnancy and rearing young children were muted at older maternal ages. We conclude that the risk for AUD mothers while pregnant or rearing small children varies substantially as a function of the age of the child, the genetic risk of the mother and the mother’s age. These risk and protective factors can interact substantially with one another.

Pregnancy is often considered a “window of opportunity” for women motivated to stop alcohol and other substance use for the health and well-being of their baby [1, 2]. Amongst the nearly half (49.4%) of U.S. women reporting pre-pregnancy alcohol use, only 6.4% continued to drink at pre-pregnancy levels. The majority (87%) quit during pregnancy, with another 6.6% cutting down on their drinking [3].

Similar patterns are seen in Sweden with reductions during pregnancy in alcohol use [4, 5] and binge drinking [6]. The protective effect of pregnancy extends to Alcohol Use Disorder (AUD), with reduced rates of AUD registration during pregnancy [7]. This pattern was seen in those at high risk for developing AUD, and particularly pronounced in younger women who became pregnant before the age of 25 [7].

From these findings, it is logical to consider the post-partum period starting with infancy, when the child is 1–12 months old. Alcohol use tends to increase during this time. Analyses of National Survey on Drug Use and Health (NSDUH) data showed past month alcohol use increased from 6.2% prenatally to 31.9% at 3 months postpartum and 52% at 11 months postpartum [8]. Changes in patterns of heavy or problem drinking during this time are less clear. While some describe the immediate postpartum period as stressful and associated with increased rates of problem drinking, others emphasize the maternal role serving as a protective factor reducing AUD risk [9]. Furthermore, breastfeeding during this time is correlated with reduced alcohol consumption [10, 11] and heavy episodic drinking [12]. In Sweden, the protective effect of pregnancy on AUD risk persists over the first and second year post-partum but declines in magnitude [7]. Studies of maternal alcohol use while children are toddlers and preschoolers are limited, but suggest that reductions in alcohol intake during pregnancy are often reversed when the child is 3–4 [13, 14].

Studies of the protective effects of pregnancy or rearing small children on AUD risk have not typically considered maternal genetic risk to AUD or age. This is an important limitation as genetic risk is amongst the strongest known risk factors for developing AUD [15] and with respect of AUD risk, pregnancy in younger women may differ substantially from pregnancies in more mature women. Swedish teenage mothers compared to women giving birth at ages 25–29, have much higher rates of unplanned pregnancy, depression ratings, pre-pregnancy binge drinking, and prenatal smoking and lower rates of self-esteem and social support [16].

In this study, using the high quality national Swedish registers and our pedigree-based index of genetic risk – the family-genetic risk score (FGRS) – we seek, by studying women in their first pregnancy and child rearing experiences, to deepen our understanding of how i) the mother’s genetic risk for AUD, ii) the mother’s age and iii) the mother’s maternal status – that is, being pregnant or raising an infant, toddler, or preschooler – act and interact in their impact on mother’s risk for AUD onset.

## Methods

We made use of the Swedish total population register to define a study population, along with other nationwide Swedish registers (Appendix Table 1) to retrieve information on the study individuals, enabled by linkage of each person’s unique identification number (for confidentiality reasons, replaced with a serial number). Ethical approval was secured from the regional ethical review board in Lund, Sweden, and personal informed consent was not required (No. 2008/409). Alcohol use diagnoses were searched for using the Swedish Hospital Discharge Register, Outpatient Care Register, almost nationwide primary care data, the Swedish Prescribed Drug Register, the Swedish Cause of Death Register, the Swedish Criminal Register, and the Swedish Suspicion Register. For a description of the AUD definition, see Appendix Table 2. Live childbirths were detected through the multi-generational register.

We found 1,797,574 female individuals born in Sweden between 1960 to 1995, alive and not having emigrated at age 18. Women with a child dying before the age of 6 months (*n* = 5577) were excluded. Individuals were followed from age of 15 until age 40, first occurrence of AUD, death, emigration, start of second pregnancy or December 31st 2018, whichever occurred first. Women with AUD onset or pregnancy start prior to age 15 were excluded from analyses, as well as women having two consecutive childbirths closer than nine months apart (*n* = 1611). We defined four childbirth related time-varying exposure variables: pregnancy (from the time point of 242 days prior to birth until birth), infant (child aged 0–12 months), toddler (13–36 months) and preschool (37–60 months). The starting time point of pregnancy was defined as in Edwards et al. [7].

For the study participants, we calculated the family genetic risk score (FGRS) for AUD. The FGRS estimates an individual’s genetic liability to AUD, making use of register-based pedigrees of first- through fifth-degree relatives, reflecting the relative lack or excess of disease, relative to population expectations. The estimate is based on morbidity risks in relatives from phenotypic data, controlling for cohabitation effects (for details, see Appendix Table 3). There is now an extensive literature using this method [17–23] and it has been shown to agree with similar statistical approaches [24, 25]. FGRS were scaled within the total cohort of men and women (n = 3,693,847), such that they had a mean of zero and standard deviation of one.

Crude age-varying AUD incidence rates (Appendix Table 4) were calculated by splitting data in age intervals 15–19, 20–24, 25–29, 30–34, and 35–39, followed by dividing the number of new cases in each age interval with the total number of person years at risk in that interval, multiplied by 10,000. These incidence rates were also derived within exposure times of pregnancy, having an infant, having a toddler, and having a preschooler. All rates were also presented in data stratified by FGRS (split at the mean level of zero).

The main analysis of the study makes use of AUD onset as the outcome, with the childbirth related variables as exposures, and especially whether there are (additive) effect modifications of these special time periods in a woman’s life by her genetic liability to AUD, as being measured by FGRS. Time to AUD onset is modelled on the additive scale, with age as time scale, by applying Lin & Ying’s additive hazards regression [26] (by using the Aalen-function in the R-package timereg [27]), being similar to Aalen’s additive hazards regression [28], but simplifying interpretations of estimated parameters by the assumption of time-constant effects instead of time-varying effects. In both models, the baseline is allowed to vary freely. That is, we here model interactions between genetic risk and the exposures of pregnancy and child rearing on an additive scale. This is because, as outlined in detail elsewhere, our main interest is the population impact of such interactions [29]. That is, does the exposure to two risk factors, in this case maternal genetic risk for AUD and exposure to pregnancy and various early stages of child development, produce greater or fewer cases of AUD than would be expected from the risk factors operating independently? This is the question additive models of interactions are designed to address.

Along with the inclusion of main and interaction effects of the child-birth related variables and FRGS, the model is corrected for birth year of the women, as a piece-wise constant effect, enabled by splitting data over time (age). As women with high FGRS are more likely to have an onset of AUD at a younger age, and are more likely to be young at first pregnancy, the confounding effect of FGRS is controlled for by the inclusion of a piece-wise constant age-varying main effect. In additional modeling, to remove possible confounding effects, we further corrected for educational attainment (as a categorical variable divided into pre-secondary, secondary and post-secondary levels) and diagnosis of major depression as a time-varying variable, with the effects of both covariates allowed to vary with age by the use of piece-wise constant age-varying effects. Both covariates are strongly linked to AUD. See Appendix Table 2 for definitions of the covariates. From the modeling, we retrieve estimates of additive effects on the hazard rate scale, presented per 10,000 person years.

After having applied Kolmogorov-Smirnov tests on all variables included in our main analysis to test for significant time-varying effects (see Appendix Table 5), with the results that all variables were varying with age, a more complex analysis was performed. The assumption of age-constant effects of the childbirth related variables were relaxed by the estimation of piece-wise constant effects of the interactions between FGRS and child-birth related variables, along with the same main effects. The twenty-five-year time span of ages at study, 15–39, were divided into five equally sized time intervals of five years each. As the combination of getting a child and having an AUD onset is uncommon amongst both younger and older individuals (see Appendix Table 4), narrower intervals would not likely have provided us any further reliable information.

Levels of significance of 0.05, 0.01, 0.001, and 0.0001 were marked in the tables. Data analysis was conducted from August 16, 2024, to January 14, 2025, and was performed using R, version 4.4.0 [30] (Appendix Table 6) and SAS, version 9.4 [31].

## Results

Table 1 provides a description of the main features of our population sample. We began with nearly 1.8 million women of whom 25,749 had an onset of AUD between the ages of 15 and 40. Of these, 195 experienced onsets during their first pregnancy and 201, 764, and 794 experienced first onset when their first child was an infant (age 0–1), toddler (ages 1–3) or preschooler (ages 4–5), respectively. As seen in the bottom row of this table, all women with AUD onset aged 15–40 had a mean FGRS_AUD_ of 0.65 which increased progressively in those with an AUD onset during pregnancy (0.76), and when raising an infant (0.80) or toddler (1.06) leveling out when raising a preschooler (0.97).

**Table 1:** Descriptive statistics of the study in terms of sample size, birth year, age at onset, age at birth, in relation to timing of pregnancy- and post pregnancy related onset of AUD.

				AUD cases with onset between ages 15–40^a^					
	All female	All parous female	All female AUD cases	All female AUD cases	All parous female AUD cases	Pregnancy AUD onset	Infant 0–1 yrs (0–12 months) AUD onset	Toddler 2–3 yrs (13–36 months) AUD onset	Preschool 4–5 yrs (37–60 months) AUD onset
Number	1,791,997	1,208,326	49,420	25,759	13,290	195	201	764	794
Year of birth, mean (sd)	1977.5 (10.59)	1974.1 (9.08)	1975.3 (10.53)	1981.0 (9.51)	1978.6 (9.05)	1981.5 (9.10)	1978.5 (9.50)	1978.6 (9.81)	1977.4 (9.10)
Age at first AUD diagnosis, mean (sd)	32.5 (11.42)	34.5 (11.63)	32.5 (11.42)	24.6 (6.21)	24.2 (6.36)	26.2 (6.03)	25.4 (5.68)	25.9 (5.03)	27.9 (4.81)
Age at first birth, mean (sd)	27.5 (4.93)	27.5 (4.93)	25.4 (5.24)	26.4 (5.32)	26.4 (5.32)	26.5 (6.00)	24.8 (5.65)	23.9 (5.01)	23.9 (4.76)
FGRS_AUD, mean (sd)	0.00 (1.00)	0.03 (1.01)	0.66 (1.48)	0.65 (1.47)	0.76 (1.52)	0.76 (1.63)	0.80 (1.38)	1.06 (1.77)	0.97 (1.52)

^a^Based on the same inclusion criteria and censoring mechanisms as used in the time to event analysis.

**Fig. 1:**
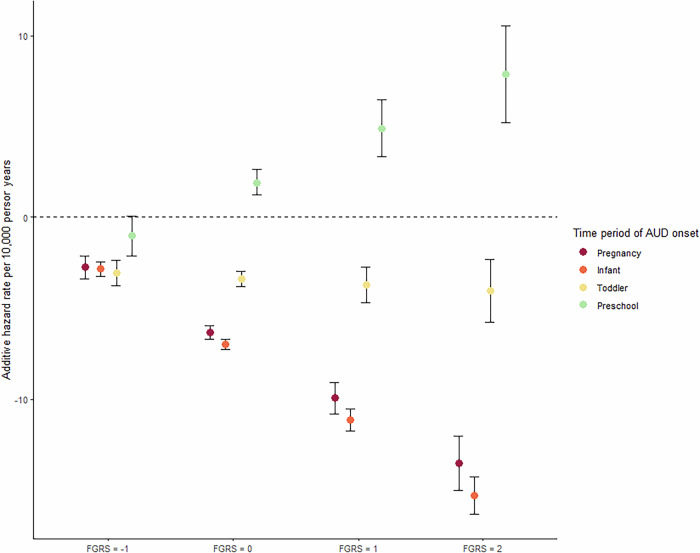
The age-constant additive hazard rates (point estimates and 95% CIs) for a first onset of Alcohol Use Disorder (AUD) during a first pregnancy, and during the raising of a first infant (aged 0–12 months), toddler (13–36 months) or preschooler (37–60 months) with FGRS_AUD_ as effect modifier. As women with high FGRS_AUD_ are more likely to have an onset of AUD at a younger age, and are more likely to be young at first pregnancy, the confounding effect of FGRS_AUD_ is controlled for by the inclusion of an age-varying main effect. The y-axis depicts the additive hazard of AUD onset per 10,000 person-years. The x-axis represents level of the standardized genetic risk for AUD, with, from left to right, levels that are low (−1 SD), mean (0), high (+1 SD) and very high (+2 SD) risk.

The results of our age-constant model are depicted in Table 2 and Fig. 1. In Table 2, we see a substantial and significant positive hazard effect on AUD risk from the FGRS_AUD_ across our age groups and a robust protective effect for being pregnant and having an infant. This protective effect declines moderately with a toddler but reverses sign and becomes significantly predisposing with a preschooler. We also see significant *negative* interactions between the genetic risk for AUD and being pregnant or having an infant. That is, being pregnant or having an infant is more protective against AUD for individuals at high versus low AUD genetic risk. No significant interaction is seen for having a toddler. By contrast, we observe a significant positive interaction between the FGRS_AUD_ and having a preschooler. That is, having a preschooler increases the risk for AUD more strongly in mothers with high versus low levels of FGRS_AUD_.

**Table 2:** Model estimates of age-constant analysis: Age-constant additive hazard rates, measured per 10,000 person years, of pregnancy, infant, toddler and preschool phases on onset of AUD, with FGRS_AUD_ as effect modifier.

	Age 15–39, n = 1,790,386, person years = 34,553,657	
	Additive hazard rate	95% CI^a^
FGRS_AUD_; 15–19 20–24 25–29 30–34 35–39	1.26 7.65 5.37 4.01 3.24	(1.19–1.33)**** (7.28–8.02)**** (5.01–5.73)**** (3.66–4.36)**** (2.90–3.58)****
Pregnancy	−6.33	(−6.71-(-)5.95)****
Infant	−6.98	(−7.27-(-)6.69)****
Toddler	−3.38	(−3.78-(-)2.98)****
Preschool	1.95	(1.25–2.65)****
FGRS_AUD_*Pregnancy	−3.59	(−4.24-(-)2.94)****
FGRS_AUD_*Infant	−4.14	(−4.58-(-)3.70)****
FGRS_AUD_*Toddler	−0.33	(−1.09–0.44)
FGRS_AUD_*Preschool	2.98	(1.82–4.14)****

As women with high FGRS_AUD_ are more likely to have an onset of AUD at a younger age, and are more likely to be young at first pregnancy, the confounding effect of FGRS_AUD_ is controlled for by the inclusion of an age-varying main effect.

^a^ Significance levels for p-values: *<0.05, **<0.01, ***<0.001, ****<0.0001.

These results are clearly seen in Fig. 1 where we depict model predictions at four levels of FGRS_AUD_: low (−1.0 SD), mean (0), high (+1 SD) and very high (+2 SD). The y-axis represents the hazard rate for AUD per 10,000 person years. Focusing first on the impact of being pregnant or having an infant, we see a substantial and similar protective effect for those at low genetic risk which becomes appreciably stronger with rising levels of FGRS_AUD_. The effect of having a toddler is moderately protective but does not change significantly as a function of AUD genetic risk. By contrast, having a preschooler has a large *positive* interaction with FGRS_AUD_ such that at low genetic risk, a preschooler is modestly protective, but becomes substantially predisposing to the development of AUD with rising levels of FGRS_AUD_.

**Fig. 2:**
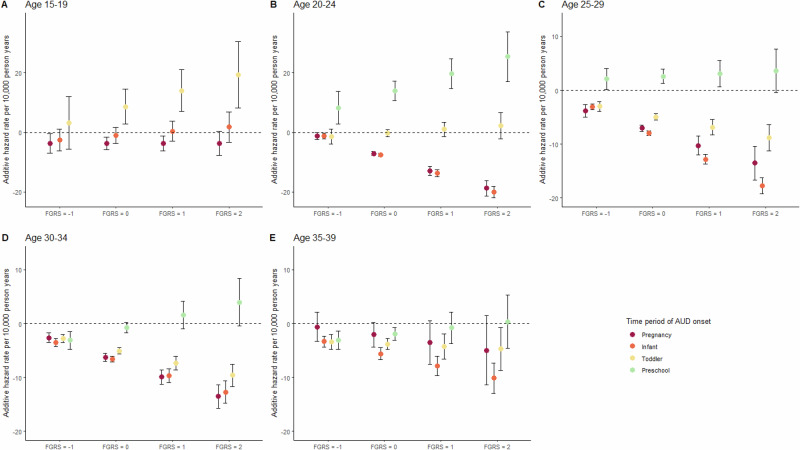
The age-varying additive hazard rates (point estimates and 95% CIs) for a first onset of Alcohol Use Disorder (AUD) during a first pregnancy, and during the raising of a first infant (aged 0–12 months), toddler (13–36 months) or preschooler (37–60 months) with FGRS_AUD_ as effect modifier for mothers of five different ages. **A** age 15–19; (**B**) age 20–24; (**C**) age 25–29; (**D**) age 30–34; (**E**) age 35–39. As women with high FGRS_AUD_ are more likely to have an onset of AUD at a younger age, and are more likely to be young at first pregnancy, the confounding effect of FGRS_AUD_ is controlled for by the inclusion of an age-varying main effect. The y-axis depicts the additive hazard of AUD onset per 10,000 person-years. The x-axis represents level of the standardized genetic risk for AUD, with, from left to right, levels that are low (−1 SD), mean (0), high (+1 SD) and very high (+2 SD) risk.

We next examine how the relationships seen in our first analysis change as a function of maternal age, divided into 5 periods: 15–19, 20–24, 25–29, 30–34 and 35–39. We depict, in Appendix Table 4, the number of AUD onsets and crude age-varying incidence rates of AUD per 10,000 person years. The number of teenage mothers with preschool children who developed AUD (*n* = 5) was too small to permit statistically stable estimates. The results of this model are detailed in Table 3 and Fig. 2. We here focus on the latter. Note that, to capture our findings in Fig. 2, we expanded, for ages 15–19 and 20–24, our y-axis from that seen in Fig. 1.

**Table 3:** Model estimates of age-varying analysis: Age-varying additive hazard rates, measured per 10,000 person years, of pregnancy, infant, toddler and preschool phases on onset of AUD, with FGRS_AUD_ as effect modifier.

	Age 15–39, n = 1,790,386, person years = 34,553,657	
	Additive hazard rate	95% CI^a^
FGRS_AUD_; 15–19 20–24 25–29 30–34 35–39	1.22 7.60 5.63 4.13 3.26	(1.15–1.29)^****^ (7.20–8.00)^****^ (5.23–6.03)^****^ (3.74–4.52)^****^ (2.90–3.62)^****^
Pregnancy; 15–19 20–24 25–29 30–34 35–39	−3.69 −7.08 −7.05 −6.26 −2.05	(−5.81-(-)1.57)^***^ (−7.77-(-)6.39)^****^ (−7.63-(-)6.47)^****^ (−6.96-(-)5.56)^****^ (−4.36–0.26)
Infant (0–1 yrs) 15–19 20–24 25–29 30–34 35–39	−1.12 −7.49 −7.94 −6.57 −5.57	(−3.81–1.57) (−8.05-(-)6.93)^****^ (−8.33-(-)7.55)^****^ (−7.10-(-)6.04)^****^ (−6.64-(-)4.50)^****^
Toddler (2–3 yrs); 15–19 20–24 25–29 30–34 35–39	8.55 −0.22 −4.94 −5.06 −3.81	(2.71–14.39)^**^ (−1.37–0.94) (−5.52-(-)4.36)^****^ (−5.62-(-)4.50)^****^ (−4.82-(-)2.80)^****^
Preschool (4–5 yrs); 15–19 20–24 25–29 30–34 35–39	^b^ 13.92 2.58 −0.74 −1.92	(10.62–17.21)^****^ (1.24–3.92)^***^ (−1.72–0.24) (−3.07-(-)0.77)^***^
FGRS_AUD_*Pregnancy; 15–19 20–24 25–29 30–34 35–39	0.02 −5.83 −3.22 −3.63 −1.45	(−1.96–2.00) (−6.96-(-)4.70)^****^ (−4.59-(-)1.85)^****^ (−4.52-(-)2.74)^****^ (−4.04–1.14)
FGRS_AUD_*Infant; 15–19 20–24 25–29 30–34 35–39	1.43 −6.26 −4.89 −3.08 −2.27	(−0.79–3.64) (−7.14-(-)5.38)^****^ (−5.52-(-)4.26)^****^ (−3.94-(-)2.22)^****^ (−3.31-(-)1.23)^****^
FGRS_AUD_*Toddler; 15–19 20–24 25–29 30–34 35–39	5.38 1.22 −1.93 −2.26 −0.44	(−0.03–10.79) (−0.92–3.36) (−2.99-(-)0.87)^***^ (−3.13-(-)1.39)^****^ (−2.12–1.23)
FGRS_AUD_*Preschool; 15–19 20–24 25–29 30–34 35–39	^b^ 5.70 0.50 2.36 1.15	(1.70–9.70)^**^ (−1.25–2.26) (0.45–4.27) (−0.93–3.23)

^a^ Significance levels for p-values: **<0.01, ***<0.001, ****<0.0001. P value threshold set at p < 0.01 to correct for multiple tests with each risk period – that is pregnancy to preschool child. ^b^Not estimated due to sparse data.

At age 15–19 (Fig. 2a), the results differ substantially from those seen in the entire sample. Pregnancy is slightly protective against AUD onset but does not vary by level of FGRS_AUD_. Having an infant is unrelated to AUD risk across levels of genetic risk. Having a toddler however, is unrelated to AUD risk in those with low levels of FGRS_AUD_ but is associated with a significantly greater risk that increases substantially with rising levels of FGRS_AUD_.

For mothers aged 20–24 (Fig. 2b), being pregnant or having an infant is protective against AUD onset but that effect is strongly dependent on level of genetic risk. By contrast, the effect of having a young toddler is unrelated to risk. Having a preschooler, by contrast, increases AUD risk substantially with that risk being much stronger with higher levels of FGRS_AUD_.

Two changes are seen in these results for mothers aged 25–29 (Fig. 2c). First, the impact of having a toddler becomes protective and, like the findings for being pregnant or having an infant, stronger in those at high genetic risk. Second, the predisposing effect of having an older toddler on AUD decreases substantially and is no longer dependent on level of FGRS_AUD._

For mothers aged 30–34 (Fig. 2d), having a preschooler is now modestly protective for those at low genetic risk for AUD but becomes unrelated to AUD risk in those with high levels of FGRS_AUD_. The protective effect of having a toddler now approaches that of being pregnant or having an infant, growing stronger with increases genetic risk.

Finally, in older mothers (where our sample size was smaller and our confidence intervals larger), pregnancy no longer had any significant effect on AUD risk. Having an infant or young toddler remained protective, with increasing effects with higher genetic risk. Having a preschooler was modestly protective only for those with low or average levels of FGRS_AUD_.

## Discussion

We here sought to first explore how genetic liability to AUD interacted with the maternal status of a first pregnancy or rearing a first child at three different developmental stages (infancy, toddlerhood and preschooler) to influence risk for AUD onset. Second, we then examined how this relationship varied over maternal age.

Our initial analyses demonstrated that, with respect to their impact on AUD risk, the 5 maternal states we examined could be divided into three groups. Being pregnant or caring for an infant had very similar effects, reducing risk for AUD onset with the impact of that reduction increasing substantially with rising levels of AUD genetic risk. That is, the higher a mother’s genetic risk for AUD, the greater was the protective effect of being pregnant or raising an infant. Having a toddler, by contrast, was for those at low genetic risk, equally protective for AUD onset as being pregnant or raising an infant. By unlike these two maternal states, having a toddler did not interact with genetic risk. Finally, for mothers at low genetic risk for AUD, raising a toddler has a slight protective effect on AUD risk. However, for those with average, high or very high genetic risk, raising a toddler was an increasing strong risk factor for AUD onset – the *opposite effect* seen with being pregnant or having an infant.

In our second set of analyses, we show that the associations between genetic risk and maternal state are quite variable across maternal age. Three broad trends are noteworthy. First, the protective effect of being pregnant or having an infant are only marginally present in teenage mothers. Second, younger mothers are more sensitive to the AUD predisposing effects of toddlers and preschoolers than older mothers. Third, the effects of all the maternal states are generally muted in the oldest maternal group (age 35–39).

Sweden has among the highest rates of breast feeding in the world [32]. Over the decades covered in this report, rates of breast feeding in the first two months after birth were around 65% in 1960 rising to over 90% by 1995 [33]. Consistent with prior evidence of the inverse association between lactation and alcohol consumption in women [10–12], the high rate of breast feeding in Sweden may contribute to the protective effects of having an infant.

Most prior positive studies of gene-environment interaction in psychiatric and drug abuse disorders have shown that high genetic liability increases the pathogenic effect of *adverse* environmental exposures leading to *increased* rates of illness [34–39]. Our results for pregnancy, and having an infant and toddler, most frequently show, by contrast, that high levels of FGRS_AUD_ augment the impact of *protective* exposures on AUD risk leading to a *reduction* in rates of illness. This finding result is not as counter-intuitive as it might appear as strong protective effects will be very hard to demonstrate for disorders whose frequency is low in those at reduced genetic risk. What is rarer is our finding that a related putative risk factor in the same research design – raising a preschooler – show the more typical effect where high genetic risk increases the risk of a naturally occurring pathogenic influence.

Our finding that AUD risk was reduced for both the pregnancy and infant periods was not surprising. The teratogenic effects of alcohol are well-known [40], prompting global public health messaging for women to avoid alcohol consumption when pregnant and while breastfeeding [41].

We did not anticipate the strong evidence for increased AUD risk in mothers with both a preschooler and high AUD genetic liability. Research on maternal drinking during the preschool periods is limited [42], but some reports suggest increased maternal drinking. One study found drinking didn’t return to pre-pregnancy levels until 3 years postpartum [14]. Another found the risk of harmful post-partum alcohol use increased from 0.7% (infant period) to 2.6% (preschool period) [13]. Significant heterogeneity has been reported in these longitudinal alcohol use patterns from conception to 5 years (preschool), with some women experiencing an escalation in risky drinking (12%), while others quit drinking entirely (10.2%) [43]. Overall, however, more women reported consuming 4 + drinks per week while raising a preschooler than prior to pregnancy. Given that women progress more rapidly from alcohol use to problems than do men [44], our findings confirm the importance of routine screening for alcohol problems in women with toddlers and preschoolers, especially if they are young and have a positive family history for AUD.

Our findings that protective effects on AUD risk of pregnancy and having young children are stronger in those with high genetic liability is consistent with a range of findings of various treatment trials [45] and epidemiological investigations such as evidence that the impact of a healthy life style on risk for cardiovascular disease is stronger in those with high genetic liability [46]. In this context, pregnancy and having young dependent children can be conceptualized as a natural “intervention” or turning point that constrains and motivates behavior change in this case involving abuse of alcohol.

## Limitations

These results should be interpreted in the context of four potentially important methodological limitations. First, our findings are dependent on the validity of the diagnoses of AUD obtained from the Swedish medical and criminal Swedish registries. Access to medical care is very widely available in Sweden and mothers who are pregnant or have small children are likely to be often seen by medical personnel who are sensitive to possible substance use problems. Registry diagnoses have a number of advantages for research, as neither cooperation effects, social desirability biases nor recall problems are of relevance. However, our results require individuals with alcohol-related problems to have contacts with medical and or legal authorities and will not, therefore, fully replicate findings from personal interviews. The validity of our AUD diagnoses is supported by the high concordance rates across ascertainment methods [47, 48] and the similarity of observed patterns of resemblance in relatives to personally interviewed samples [49, 50].

Second, causal inference in epidemiological samples like ours cannot be assumed and so our results should be interpreted with some caution. We performed co-sibling analyses of the protective effect on AUD onset of pregnancy and having an infant, toddler or preschooler (Appendix Table 7) demonstrating that the first two of these associations were entirely causal and the second two had modest degrees of familial confounding.

Third, our main analyses did not consider the role of fathers. Follow-up analyses showed that compared to families with cohabiting fathers, in those where the mothers were not living with the father (details Appendix Table 8), the protective effect of pregnancy and having a toddler on AUD risk was reduced, the protective effect of having an infant was unchanged and the predisposing effect of having a preschooler was strengthened.

Fourth, our analyses excluded effects of second childbirths, and also mothers were censored at the time of the second pregnancy, and could not contribute to e.g. effect of preschool phase for the first child. This may have produced a bias, especially when we divided our sample by age. We therefore removed the exclusion and allowed the second childbirth to contribute to the estimated effects of maternal states, in the way that a woman with a pregnancy and a toddler at once would contribute to both states at that time point. Mothers were censored at their third pregnancy onset. As seen in Appendix Fig. 1, our results were minimally changed, with a slightly smaller predisposing effect of having preschoolers.

Fifth, we did not control for many covariates in our main analyses because of concerns of over-control. To examine the sensitivity of our findings to key covariates, we repeated our analyses controlling for two of the most obvious: maternal major depression (MD) and maternal educational attainment (EA). In a modified Table 1 presented in Appendix Table 9, we show that rates of MD in our AUD mothers were nearly three times that seen in the general population and the proportion with the lowest level of EA in Sweden (not completing secondary education) was nearly five times greater. The main changes, seen in Appendix Fig. 2 and Table 10, is a shift in the impact of having a preschool child which is now protective in mothers at low genetic risk, has no impact on risk in those at average risk and the increased risk in those with high genetic liability is modestly attenuated. So, reassuringly, even the inclusion of these covariates strongly associated with maternal AUD risk produce only modest changes in our findings.

Sixth, our findings are limited to the Swedish population which has a particular set of social and health care policies that could have impacted on our results.

## Conclusions

Understanding the etiology of psychiatric and substance use disorders likely requires research designs which integrate, over development, the actions and interactions of genetic and environmental risk factors. We utilize such a design and illustrate the potential complexity of the findings in the following conclusions. First, the maternal states examined have diverse impacts on risk for AUD. Being pregnant is consistently protective across levels of genetic risk and almost all ages. Having an infant is protective in all but teenage mothers. However, raising a preschooler has widely variable effects, strongly predisposing in mothers aged 20–24, modestly predisposing in mothers 25–29, but protective in some older mothers. Second, we see many examples of gene-environment interactions, where we define environment broadly to include the state of pregnancy. Most strikingly, across our entire sample, being pregnant and raising an infant are protective for those at high genetic risk, while the opposite effect is seen raising a preschooler. Third, we see major shifts in the impact of maternal states and genetic risk across developmental periods. On average, the effects of maternal states on AUD risk decrease in magnitude with increasing maternal age. An illustration of the potential complexity of our findings is the following three-way interaction between genetic risk, maternal state and maternal age. In teenage pregnancies, having a toddler predisposes to MD risk and this becomes stronger at high genetic risk. But in mothers age 30–34, a toddler is associated with a protective effect that grows stronger at high genetic risk. We doubt that such complexities will arise in risk prediction for all psychiatric and substance use disorders, but suggest that our field should encourage research designs that have the capacity to detect the variable impact of environmental risk and genetic risk over critical developmental periods.

## Supplementary information

Appendix

## Data Availability

The data for this study are not publicly available due to legal restrictions with regard to the nationwide Swedish registers, but they can be acquired directly from the responsible authorities pending their approval. Kristina Sundquist MD PhD had full access to all the data in the study and takes responsibility for the integrity of the data and the accuracy of the data analysis.
