# Supplementary material for: The relationship in women between genetic liability and the risk of onset of alcohol use disorder while pregnant or rearing an infant, toddler, or preschool child
Source: Mol Psychiatry. 2025 Dec 14;31(5):2481–8. doi: 10.1038/s41380-025-03410-5 (PMC13099424; doi:10.1038/s41380-025-03410-5)

Supplementary Material

TABLE 1 – Description of Registers

*Total Population Register*

The Total Population Register was created digitally in 1968 and includes yearly information, from the National Tax Board, on individuals registered in Sweden. It is possible to retrieve information on e.g. birth, death, immigration, emigration, migration within Sweden, place of residence, family information, civil status, etc.

*Multi-Generation Register*

The Multi-Generation Register is a register made up of persons who have been registered in Sweden at some time since 1961 and those who were born in 1932 or later. These are called index persons. The register contains connections between index persons and their biological parents. In 2024, more than 11 million index persons were included in the register. The Multi-Generation Register is a part of the register system for Total Population Register, where information comes from the National Tax Board. Every year, a new version of the register is created, including new index persons who immigrated or were born during the year. Information from the Multi-Generation Register may be disclosed for research and statistical purposes. See https://www.scb.se/vara-tjanster/bestall-data-och-statistik/register/flergenerationsregistret/

*National Patient Register*

In the 1960's the National Board of Health and Welfare started to collect information regarding in-patients at public hospitals, the National Patient Register (NPR). Initially it contained information about all patients treated in psychiatric care and approximately 16 percent of patients in somatic care. The register at that time covered six of the 26 county councils in Sweden. In 1984, the Ministry of Health and Welfare together with the Federation of County Councils decided a mandatory participation for all county councils. From 1987, NPR includes all in-patient care in Sweden. Since 2001, the register also covers outpatient doctor visits including day surgery and psychiatric care from both private and public caregivers. For more information, see *https://www.socialstyrelsen.se/en/statistics-and-data/registers/national-patient-register/*

*Primary Care Data*

We also used information from our new Primary Care research dataset including individual-level information on clinical diagnoses from primary health care centers from the following Swedish counties: Blekinge (2009-2018), Dalarna (2005-2018), Gotland (2011-2018), Gävleborg (2010-2018), Halland (2007-2018), Jönköping (2008-2018), Kalmar (2007-2018), Kronoberg (2006-2018), Norrbotten (2001-2018), Skåne (1989-2018), Stockholm (2003-2018), Södermanland (1992-2018), Uppsala (2005-2018), Västra Götaland (2000-2018), Värmland (2005-2018), Västerbotten (1991-2018), Västernorrland (2008-2018), Västmanland (2014-2018), Östergötland (1990-2018), and Örebro (2006-2018). The retrieval of data differs due to timing of digitalization of patient records. In 2018, 99% of the Swedish population lived in these 20 counties. For more information see *Sundquist, J., Ohlsson, H., Sundquist, K. et al. Common adult psychiatric disorders in Swedish primary care where most mental health patients are treated. BMC Psychiatry 17, 235 (2017).*

*The Population and Housing Censuses*

Every fifth year between 1960 and 1990 Sweden conducted censuses. These registers include among other things, the population's employment, educational level, the composition of households and housing. *For more information, see https://www.scb.se/en/finding-statistics/statistics-by-subject-area/population-and-living-conditions/population-composition-and-development/population-and-housing-census-1960-1990-tpr/*

*Prescribed Drug Register*

The Swedish Prescribed Drug Register started in July 2005 and includes all prescribed drugs being fetched at pharmacies, linked to personal numbers. See *https://www.socialstyrelsen.se/en/statistics-and-data/registers/national-prescribed-drug-register/*

*Cause of Death Register*

The Cause of Death Register includes all deaths occurring in Sweden from 1961 (including for Swedish citizens dying abroad) and is updated yearly. There is also a historical register between the years 1952 to 1960. For more information, see *https://www.socialstyrelsen.se/en/statistics-and-data/registers/national-cause-of-death--register/*

*Criminal and Suspicion Register*

The Swedish Criminal Register and the Swedish Suspicion Register includes individual-level information on all committed crimes from 1973 and all suspicions of crimes related to an individual from 1998. For more information, see *https://polisen.se/lagar-och-regler/behandling-av-personuppgifter/polisens-register/*

*Longitudinal integrated database for health insurance and labour market studies (LISA)*

LISA was created by Statistics Sweden in 1990 and includes yearly information on e.g. employment, educational level and disposable income for individuals (from age 16) registered in Sweden. See *https://www.scb.se/vara-tjanster/bestall-data-och-statistik/register/lisa/*

TABLE 2 – Definition of Disorders

| Disorder | Registers Used | Definition |
| --- | --- | --- |
| Alcohol Use Disorder  (AUD) | Hospital Discharge Register;  Outpatient Care Register;  Primary Care Data;  Prescribed Drug Register;  Cause of Death Register;  Criminal Register;  Suspicion Register | Alcohol Use Disorder (AUD) was identified in the Swedish medical and mortality registries by ICD codes: ICD8: 571.0, 291, 303; ICD9: V79B, 305A, 357F, 571A-D, 425F, 535D, 291, 303; ICD 10: E24.4, G31.2, G62.1, G72.1, I42.6, K29.2, K70, K85.2, K86.0, O35.4, F10.1-F10.9; in the Swedish Criminal Register and the Swedish Suspicion Register with at least two registrations of drunk driving (suspicion code 3005, law 1951:649 (paragraph 4 and 4A)) or drunk in charge of a maritime vessel (suspicion code 3201, law 1994:1009 (chapter 20, paragraph 4 and 5)); in the Prescribed Drug Register by the drugs disulfiram (Anatomical Therapeutic Chemical (ATC) Classification System N07BB01), acamprosate (N07BB03), and naltrexone (N07BB04). |
| Major Depression  (MD) | Hospital Discharge Register;  Outpatient Care Register; Primary Care Data | Major Depression (MD) was identified in the Swedish medical registries by ICD codes: ICD8: 296.0, 296.2, 298.0, 300.4; ICD9: 296B, 298A, 300E; ICD10: F32, F33. |
| Educational attainment | The Population and Housing Censuses; LISA | Highest educational attainment was categorized into levels of 1: Pre-secondary education, 2: Secondary education and 3: Post-secondary education. |

TABLE 3 – Family Genetic Risk Score derivation

| The dataset for the calculations includes:  Column1 = Identification number of the proband (Born 1932-1995)  Column2 = Identification number of the relative (1st to 5th degree relatives)  Column3 = Proportion of shared additive genetic effects (0.03125 to 0.50) with the proband  Column4 = Year of Birth of relative  Column5 = Sex of relative  Column6 = Age at registration for trait  Column7 = Age at end of follow-up (2018-12-31 or age at death, or age at emigration whichever came first) |
| --- |
| **Step 1**: Using all unique relatives with a registration for the disorder, we non-parametrically estimated the distribution of *Age at first registration*. The empirical distribution is used to obtain weights for relatives without a registration for the disorder, in order to account for the proportion of the time-at-risk period they had completed at the end of follow-up. For example, for relatives at age x at end of follow-up, the weight corresponds to the proportion of relatives registered for the trait that had been registration at age x. For relatives born prior to 1958 we subtracted age at the end of follow-up with the following formula: 1958 - Year of birth of relative. This modification was done in order to control for registration effects (i.e, most registers in Sweden start in 1973 suggesting that relatives from early birth cohorts do not have the possibility to be registered at younger ages). Note that all relatives with the disorder are weighted one. |
| **Step 2**: Transform the binary variable (trait yes/no) into a z-score based on the threshold for each trait. The underlying liability of the individual is not assessable. Instead we estimated the mean of the underlying liability to obtain sex and birth decade specific Z-scores for relatives with the trait registration and relatives without the trait. We generate n random numbers from a N (0, 1) distribution and estimate the mean for relatives registered with the disorder (i.e., mean of the observations above the threshold) and for relatives without a registration (i.e., mean of all observation below the threshold). The thresholds are calculated for each decade of birth and sex. |
| **Step 3**: Correct for cohabitation effects. To estimate the cohabitation effect (i.e. “shared environment”), we created a database with all individuals in the Swedish population born in Sweden 1955-1990. We also included the number of years, during ages 0-15, that individuals resided in the same household as their biological father. We thereby were able to define two kinds of families i) “not-lived-with” father families (offspring never resided for more than 1 year in the same household or in the same community as their biological father); ii) “lived-with” father (offspring resided a minimum of 13 year in the same household as their biological father. We performed a logistic regression model with the binary trait in offspring as outcome and the binary trait in father, type of father, and their interaction as predictors. We used the interaction term as the difference of effect between genes only and genes + environment. The same approach was performed for half-siblings where we compared those who were reared together versus reared apart. The following interaction terms were used in the calculations:   \|  \| Parent/Children \| Siblings \| \| --- \| --- \| --- \| \| AUD \| 0.99 \| 0.69 \| |
| **Step 4**: Calculate the product for each relative using the four components:   1. Z-score (reflecting sex and year of birth adjusted rates) 2. Weight (reflecting the proportion of risk period they had completed) 3. Cohabitation effects 4. Proportion of shared genetic effects (0.03125 – 0.5) with the proband |
| **Step 5**: Average the product calculated in step 4 across all relatives to a proband |
| **Step 6**: Correct for the number of relatives. We multiplied the results from step 5 with a shrinkage factor. Shrinkage factor (SF): B/(B+A/C). It produces more shrinkage if B and C are small and A is large.   1. the variance of the z-score of the disorder across all relatives, 2. the variance in the mean z-score across all probands, 3. the weighted number of relatives for each proband (sum of Column 3 across each proband). |
| **Step 7**: Correct for difference by year of birth and county differences. There are 21 counties in Sweden. For each proband we used the county they had resided in during the maximum number of years (measured from 1969 and onwards) We standardized (based on the total population) the risk score by year of birth and county of the proband into a z-score with mean 0 and SD 1. This was then used as the FGRS in the analyses. |

TABLE 4 – Descriptive table with number of AUD onsets and crude age-varying incidence rates of AUD per 10,000 person years

|  |  | Timing of AUD onset | | | | |
| --- | --- | --- | --- | --- | --- | --- |
| FGRS | Age of woman | All onsets | Pregnancy-onset | Infant-onset (0-1) | Toddler-onset (2-3) | Preschool-onset (4-5) |
| All | 15-19 | 6,913; 7.74 | 29; 5.71 | 42; 9.44 | 53; 22.35 | ^a^ |
|  | 20-24 | 8,631; 10.09 | 66; 2.88 | 71; 2.39 | 347; 11.89 | 258; 29.33 |
|  | 25-29 | 4,987; 6.95 | 51; 1.72 | 41; 0.93 | 204; 3.87 | 282; 12.16 |
|  | 30-34 | 2,959; 5.44 | 25; 1.51 | 31; 1.07 | 104; 2.28 | 171; 6.28 |
|  | 35-39 | 2,269; 5.09 | 24; 5.44 | 16; 1.91 | 56; 3.27 | 78; 4.69 |
| < 0 | 15-19 | 2,801; 4.87 | 9; 4.05 | 12; 6.50 | 16; 17.27 | ^a^ |
|  | 20-24 | 3,342; 6.00 | 20; 1.51 | 27; 1.63 | 94; 6.27 | 57; 14.49 |
|  | 25-29 | 2,001; 4.20 | 15; 0.75 | 16; 0.55 | 57; 1.72 | 91; 7.05 |
|  | 30-34 | 1,197; 3.32 | 14; 1.21 | 13; 0.64 | 42; 1.35 | 47; 2.70 |
|  | 35-39 | 981; 3.36 | 14; 4.47 | 7; 1.18 | 23; 1.93 | 31; 2.77 |
| > 0 | 15-19 | 4,112; 12.94 | 20; 6.99 | 30; 11.52 | 37; 25.61 | ^a^ |
|  | 20-24 | 5,289; 17.76 | 46; 4.76 | 44; 3.35 | 253; 17.82 | 201; 41.32 |
|  | 25-29 | 2,986; 12.37 | 36; 3.69 | 25; 1.68 | 147; 7.52 | 191; 18.57 |
|  | 30-34 | 1,762; 9.60 | 11; 2.22 | 18; 2.06 | 62; 4.29 | 124; 12.64 |
|  | 35-39 | 1,288; 8.38 | 10; 7.79 | 9; 3.67 | 33; 6.29 | 47; 8.61 |
| ^a^Number of cases ≤ 5. | | | | | | |

The crude age-varying incidence rates were calculated by splitting data in age intervals, followed by dividing the number of new cases in each age interval with the total number of person years at risk in that interval, multiplied by 10,000. These incidence rates were also derived within exposure times of pregnancy, having an infant, having a toddler and having a preschooler. All rates are also presented stratified by FGRS (splitted at the mean level of 0).

TABLE 5 – Tests of significant time-invariant effects

|  | Age 15-39, n=1,790,386, person years=34,553,657 |
| --- | --- |
|  | p-values from tests of time-invariant effects (Kolmogorov-Smirnov) |
| FGRS_AUD_ | 0.005 |
| Pregnancy | 0.006 |
| Infant | 0.007 |
| Toddler | 0.005 |
| Preschool | 0.005 |
| FGRS_AUD_*Pregnancy | 0.003 |
| FGRS_AUD_*Infant | 0.004 |
| FGRS_AUD_*Toddler | 0.002 |
| FGRS_AUD_*Preschool | 0.005 |

Applying a Holm-Bonferroni correction for multiple testing, with a significance level of 0.05, meaning that the lowest p-value was compared to 0.05/9, the second lowest to 0.05/8, etc., all tests were significant. This means that all child-birth related variables and FGRS, along with their interaction effects, are significantly time-invariant. There is evidence for that modelling the variables with age-varying effects on AUD onset could be preferable.

TABLE 6 – Details on R-packages used in statistical analyses

1. Therneau T. survival: A package for Survival Analysis in R. R package. 2024.
2. Wickham H. ggplot2: Elegant Graphics for Data Analysis. New York, NY: Springer-Verlag; 2016.
3. Wickham H, Miller E, Smith D. haven: Import and Export 'SPSS', 'Stata' and 'SAS' Files. R package. 2023.
4. Wickham H, François R, Henry L, Müller K, Vaughan D. dplyr: A Grammar of Data Manipulation. R package. 2023.
5. Barrett T, Dowle M, Srinivasan A, Gorecki J, Chirico M, Hocking T. data.table: Extension of ‘data.frame’. R package. 2024.
6. Scheike TH, Martinussen T. timereg: Dynamic Regression models for survival data. R package. 2024.
7. Neuwirth E. RColorBrewer: ColorBrewer Palettes. R package. 2022.
8. Kassambara A. ggpubr: ‘ggplot2’ Based Publication Ready Plots. R package. 2023.
9. Wickham H. stringr: Simple, Consistent Wrappers for Common String Operations. R package. 2023.

TABLE 7 – Co-sibling analyses of full siblings, with effect of pregnancy along with infant, toddler and preschool phases on the hazard ratios of AUD onset, from two versions of stratified cox regression models; an age-constant effect of women, and stratified into age at birth <=25 or >25. Women exposed to pregnancy, infant, toddler and preschool phases were age-matched to one full sibling each, as well as age- and year of birth-matched to one general population control each, not being pregnant before the age of when the exposed sibling had a child of age 5 (i.e. end of preschool phase). Exposed women had age at first birth being 15-35 for being able to compare the same sample of women across different child age categories. Analyses were controlled for birth year of the women (in sisters). Observe that censoring occurs earlier on for the exposed women due to start of a second pregnancy.

|  |  | Pregnancy AUD onset | Infant 0-1 yrs (0-12 months) AUD onset | Toddler 2-3 yrs (13-36 months) AUD onset | Preschool 4-5 yrs (37-60 months)  AUD onset |
| --- | --- | --- | --- | --- | --- |
| Exposed sisters | Number matched with full siblings  Number matched with general population controls | 204,278  294,278 | 204,091 204,093 | 186,462  186,445 | 88,458  88,540 |
|  | Person years at risk during exposure  Matched with full siblings  Matched with general population controls | 135,339.2  135,339.2 | 199,428.7 199,428.4 | 258,296.4 258,298.8 | 143,288.5  143,409.9 |
|  | Number of AUD onsets  Matched with full siblings  Matched with general population controls | 32  32 | 39  39 | 136  137 | 146  147 |
| Control sisters | Number | 204,278 | 204,091 | 186,462 | 88,458 |
|  | Person years at risk during exposure | 135,305.2 | 203,975.4 | 372,518.3 | 176,758.0 |
|  | Number of AUD onsets | 147 | 210 | 373 | 153 |
| Controls from general population matched by year of birth and age | Number | 204,278 | 204,093 | 186,445 | 88,540 |
|  | Person years at risk during exposure | 135,297.9 | 203,976.5 | 372,520.5 | 176,941.0 |
|  | Number of AUD onsets | 144 | 225 | 339 | 126 |
| Age-constant model | HR^a^ (95% CI)  Full sister controls  General population controls | 0.23 (0.16-0.34)****  0.22 (0.15-0.33)**** | 0.20 (0.14-0.28)**** 0.17 (0.12-0.25)**** | 0.49 (0.40-0.60)**** 0.61 (0.49-0.76)**** | 1.13 (0.89-1.43) 1.63^b^ (1.26-2.12)*** |
| Age at birth <= 25 | HR^a^ (95% CI)  Full sister controls  General population controls | 0.23 (0.14-0.39)****  0.42 (0.25-0.72)** | 0.23 (0.15-0.36)****  0.35 (0.22-0.54)**** | 0.69 (0.53-0.89)**  1.18 (0.89-1.57) | 1.41 (1.06-1.88)* 3.14 (2.16-4.57)**** |
| Age at birth > 25 | HR^a^ (95% CI) Full sister controls  General population controls | 0.21 (0.11-0.40)****  0.13 (0.07-0.24)**** | 0.14 (0.08-0.26)****  0.09 (0.05-0.15)**** | 0.26 (0.17-0.38)****  0.24 (0.16-0.35)**** | 0.67 (0.43-1.05)  0.63 (0.41-0.97)* |
| ^a^Significance levels for p-values: *<.05, **<.01, ***<.001, ****<.0001 ^b^Adjustment for FGRS_AUD_ gives the result 1.48 (1.23-1.77)** | | | | | |

TABLE 8 - Model estimates of analysis including living with father of child: Age-constant additive hazard rates, measured per 10,000 person years, of pregnancy, infant, toddler and preschool phases on onset of AUD, with FGRS_AUD_ as effect modifier. As women with high FGRS_AUD_ are more likely to have an onset of AUD at a younger age, and are more likely to be young at first pregnancy, the confounding effect of FGRS_AUD_ is controlled for by the inclusion of an age-varying main effect.

|  | Age 15-39, n=1,790,386, person years=34,553,657 | | | |
| --- | --- | --- | --- | --- |
| Model | Main model (multivariable) | | Model including whether mother lived with father of child (multivariable) | |
|  | Additive hazard rate | 95% CI^a^ | Additive hazard rate | 95% CI^a^ |
| FGRS_AUD_; 15-19  20-24  25-29  30-34  35-39 | 1.26  7.65 5.37 4.01 3.24 | (1.19-1.33)**** (7.28-8.02)**** (5.01-5.73)**** (3.66-4.36)**** (2.90-3.58)**** | 1.14  7.56 5.32 3.97 3.20 | (1.07-1.21)**** (7.19-7.93)**** (4.96-5.68)**** (3.62-4.32)**** (2.86-3.54)**** |
| Pregnancy^b^ | -6.33 | (-6.71-(-)5.95)**** | -3.71 | (-6.24-(-)1.18)** |
| Infant^b^ | -6.98 | (-7.27-(-)6.69)**** | -7.64 | (-9.36-(-)5.92)**** |
| Toddler^b^ | -3.38 | (-3.78-(-)2.98)**** | -0.49 | (-2.41-1.43) |
| Preschool^b^ | 1.95 | (1.25-2.65)**** | 5.37 | (2.74-8.00)**** |
| FGRS_AUD_*Pregnancy | -3.59 | (-4.24-(-)2.94)**** | -3.78 | (-4.42-(-)3.14)**** |
| FGRS_AUD_*Infant | -4.14 | (-4.58-(-)3.70)**** | -4.24 | (-4.66-(-)3.82)**** |
| FGRS_AUD_*Toddler | -0.33 | (-1.09-0.44) | -0.60 | (-1.35-0.16) |
| FGRS_AUD_*Preschool | 2.98 | (1.82-4.14)**** | 2.64 | (1.49-3.79)**** |
| Nulli-parous (reference NLW) | NA | NA | -5.88 | (-6.26-(-)5.50)**** |
| LW (reference NLW) | NA | NA | -6.98 | (-7.34-(-)6.62)**** |
| LW*Pregnancy | NA | NA | -2.63 | (-5.18-(-)0.08)* |
| LW*Infant | NA | NA | 0.98 | (-0.75-2.71) |
| LW*Toddler | NA | NA | -3.17 | (-5.13-(-)1.21)** |
| LW*Preschool | NA | NA | -4.32 | (-7.06-(-)1.58)** |
| ^a^Significance levels for p-values: *<.05, **<.01, ***<.001, ****<.0001  ^b^In model 2, these are the effects of pregnancy, etc. for the group of women not living with the biological father of their first child.  Abbreviations: LW = lived with biological father of first child some time during -3 to 3 years before birth of child. NLW = Opposite of LW (women with a first child only, i.e. not applicable to nulli-parous women). | | | | |

|  |  |  |  | AUD cases with onset between ages 15-40^a^ | | | | | |
| --- | --- | --- | --- | --- | --- | --- | --- | --- | --- |
|  | All female | All parous female | All female AUD cases | All female AUD cases | All parous female AUD cases | Pregnancy AUD onset | Infant 0-1 yrs (0-12 months) AUD onset | Toddler 2-3 yrs (13-36 months) AUD onset | Preschool 4-5 yrs (37-60 months)  AUD onset |
| Number | 1,791,997 | 1,208,326 | 49,420 | 25,759 | 13,290 | 195 | 201 | 764 | 794 |
| Year of birth,  mean (sd) | 1977.5 (10.59) | 1974.1 (9.08) | 1975.3 (10.53) | 1981.0 (9.51) | 1978.6 (9.05) | 1981.5 (9.10) | 1978.5 (9.50) | 1978.6 (9.81) | 1977.4 (9.10) |
| Age at first AUD diagnosis,  mean (sd) | 32.5 (11.42) | 34.5 (11.63) | 32.5 (11.42) | 24.6 (6.21) | 24.2 (6.36) | 26.2 (6.03) | 25.4 (5.68) | 25.9 (5.03) | 27.9 (4.81) |
| Age at first birth, mean (sd) | 27.5 (4.93) | 27.5 (4.93) | 25.4 (5.24) | 26.4 (5.32) | 26.4 (5.32) | 26.5 (6.00) | 24.8 (5.65) | 23.9 (5.01) | 23.9 (4.76) |
| FGRS_AUD,  mean (sd) | 0.00 (1.00) | 0.03 (1.01) | 0.66 (1.48) | 0.65 (1.47) | 0.76 (1.52) | 0.76 (1.63) | 0.80 (1.38) | 1.06 (1.77) | 0.97 (1.52) |
| Fraction with MD | 0.23 | 0.23 | 0.62 | 0.61 | 0.60 | 0.57 | 0.57 | 0.68 | 0.66 |
| Fraction with MD prior to AUD | NA | NA | 0.35 | 0.30 | 0.24 | 0.28 | 0.19 | 0.32 | 0.32 |
| Fraction with MD prior to first pregnancy | NA | 0.05 | NA | NA | 0.27 | 0.25 | 0.14 | 0.17 | 0.11 |
| Fraction with AUD prior to first pregnancy | NA | 0.01 | NA | NA | 0.69 | NA | NA | NA | NA |
| Educational attainment, fraction  1 = pre-secondary  2 = secondary  3 = post-secondary | 1: 0.06  2: 0.41  3: 0.52 | 1: 0.06  2: 0.43  3: 0.51 | 1: 0.18  2: 0.50  3: 0.31 | 1: 0.21  2: 0.48  3: 0.30 | 1: 0.22  2: 0.49  3: 0.29 | 1: 0.23  2: 0.50  3: 0.27 | 1: 0.29  2: 0.45  3: 0.26 | 1: 0.30  2: 0.46  3: 0.24 | 1: 0.28  2: 0.51  3: 0.20 |
|  | | | | ^a^Based on the same inclusion criteria and censoring mechanisms as used in the time to event analysis. | | | | | |

Table 9 - Descriptive statistics of the study in terms of sample size, birth year, age at onset, age at birth, in relation to timing of pregnancy- and post pregnancy related onset of AUD including covariates for prior Major Depression (MD) and educational attainment.

TABLE 10 – Model estimates of age-constant analysis including additional covariate adjustment of MD and educational attainment: Age-constant additive hazard rates, measured per 10,000 person years, of pregnancy, infant, toddler and preschool phases on onset of AUD, with FGRS_AUD_ as effect modifier. As women with high FGRS_AUD_ are more likely to have an onset of AUD at a younger age, and are more likely to be young at first pregnancy, the confounding effect of FGRS_AUD_ is controlled for by the inclusion of an age-varying main effect.

|  | Age 15-39, n=1,790,386,  person years=34,553,657 | |
| --- | --- | --- |
|  | Additive hazard rate | 95% CI^a^ |
| FGRS_AUD_; 15-19 20-24 25-29 30-34 35-39 | 1.01 6.20 4.49 3.47 2.85 | (0.94-1.08)**** (5.84-6.56)**** (4.14-4.85)**** (3.12-3.81)**** (2.51-3.19)**** |
| Pregnancy | -6.46 | (-6.84-(-)6.08)**** |
| Infant | -7.23 | (-7.52-(-)6.93)**** |
| Toddler | -4.11 | (-4.52-(-)3.71)**** |
| Preschool | 0.48 | (-0.22-1.19) |
| FGRS_AUD_*Pregnancy | -3.55 | (-4.20-(-)2.90)**** |
| FGRS_AUD_*Infant | -4.11 | (-4.55-(-)3.68)**** |
| FGRS_AUD_*Toddler | -0.39 | (-1.15-0.37) |
| FGRS_AUD_*Preschool | 2.80 | (1.64-3.96)**** |
| ^a^Significance levels for p-values: *<.05, **<.01, ***<.001, ****<.0001 | | |

Figure 1 – Effects (point estimates and 95% CIs) of First and Second pregnancies Combined, Censoring Women at start of their Third and not Second Pregnancy as in the Main Analyses
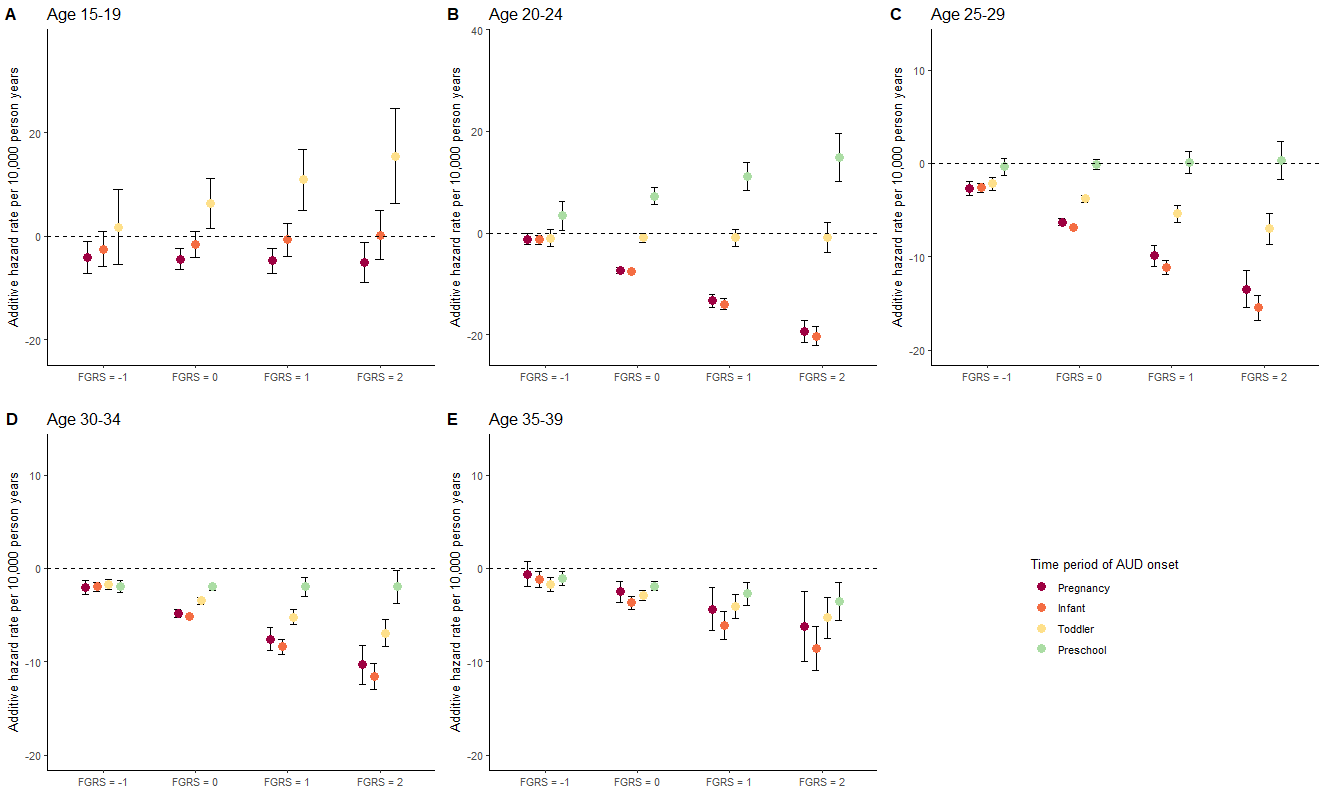


Figure 2 - The age-constant additive hazard rates (point estimates and 95% CIs) for a first onset of Alcohol Use Disorder (AUD) during a first pregnancy, and during the raising of a first infant (aged 0-12 months), toddler (13-36 months) or preschooler (37-60 months) with FGRS_AUD_ as effect modifier. Additional covariate adjustment of MD and educational attainment are included. As women with high FGRS_AUD_ are more likely to have an onset of AUD at a younger age, and are more likely to be young at first pregnancy, the confounding effect of FGRS_AUD_ is controlled for by the inclusion of an age-varying main effect. The y-axis depicts the additive hazard of AUD onset per 10,000 person-years. The x-axis represents level of the standardized genetic risk for AUD, with, from left to right, levels that are low (-1 SD), mean (0), high (+1 SD) and very high (+2 SD) risk.


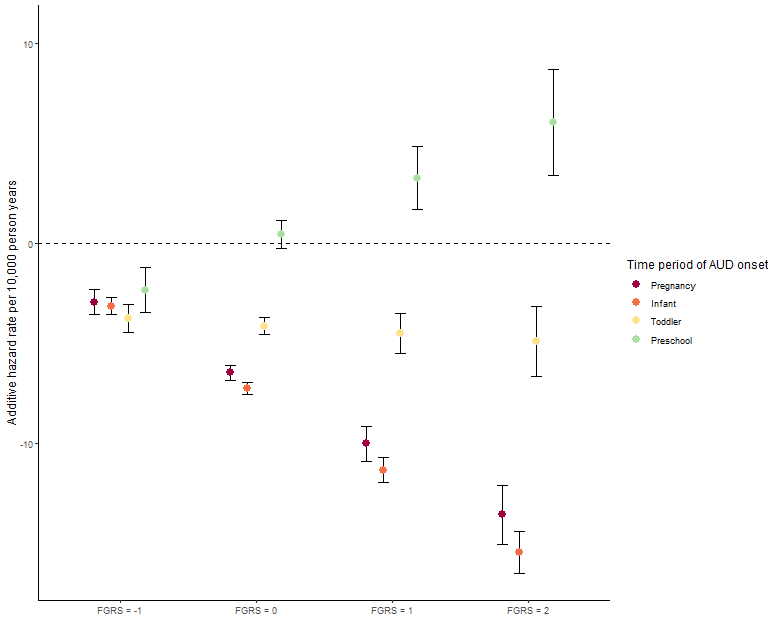

Supplement: Supplementary file 1 — Appendix [file 41380_2025_3410_MOESM1_ESM.docx]
